# Bradycardia at the onset of pulseless electrical activity arrests in hospitalized patients is associated with improved survival to discharge

**DOI:** 10.1016/j.heliyon.2020.e03491

**Published:** 2020-02-28

**Authors:** Dan Nguyen, Patricia A. Kritek, Sheryl A. Greco, Jordan M. Prutkin

**Affiliations:** a University of Washington, Internal Medicine Residency, USA; b University of Washington, Division of Pulmonary, Critical Care and Sleep Medicine, USA; c University of Washington, Divisions of Critical Care and Cardiology, Patient Care Services, USA; d University of Washington, Division of Cardiology, USA

**Keywords:** Cardiology, Cardiovascular system, Circulatory system, Critical care, Clinical research, Pulse electrical activity, Bradycardia, Cardiac arrest

## Abstract

**Background:**

Recent studies have suggested that the incidence of in-hospital pulseless electrical activity (PEA) arrests is increasing. Bradycardia in patients with in-hospital PEA is common but it is unknown if it is associated with respiratory arrest or patient outcomes.

**Objective:**

To determine risk factors and outcomes associated with bradycardic-PEA arrests, and relationship between bradycardia and respiratory arrest.

**Methods:**

This was a retrospective cohort study of all inpatient cardiac arrests at an academic medical center over a four-year period. Patient demographics, comorbidities, vital signs, arrest event data, and outcomes were abstracted from the medical record. PEA arrest was defined as a non-shockable rhythm with loss of pulse requiring cardiopulmonary resuscitation and having organized electrocardiographic activity. Bradycardia was classified as a HR < 60 bpm at the time of pulse loss. The primary outcomes were survival of arrest and survival to hospital discharge.

**Results:**

Between July 2013 and August 2017, there were 176 in-hospital patients with PEA arrests. While 105 (59.7%) survived the arrest, only 38 (21.6%) survived to discharge. A total of 66 (37.5%) were bradycardic-PEA arrests. Patients with bradycardic PEA arrests were no more likely to have their arrest precipitated by respiratory failure than non-bradycardic PEA patients (36.4% vs 27.3%, P = 0.24), but patients with non-bradycardic PEA arrests were more likely to have a CIED than non-bradycardic PEA patients (14.5% vs 3.0%, P = 0.02). On multivariate analysis, bradycardic PEA was associated with improved survival to hospital discharge (OR = 3.31, 95% CI: 1.41–7.79, p = 0.006), but not survival of arrest (OR 1.45, 95% CI: 0.68–3.09, p = 0.34). Respiratory arrest was an independent predictor of survival of code (OR 2.62, 95% CI: 1.36–5.47, P = 0.01) and to hospital discharge (OR 3.47, 95% CI: 1.35–8.91, P = 0.01). Other predictors of survival to discharge include history of coronary artery disease, and non-use of epinephrine, atropine, and sodium bicarbonate.

**Conclusion:**

In a retrospective study of hospitalized patients in the intensive care unit and non-intensive care, bradycardia at the time of PEA cardiac arrest was associated with improved survival to hospital discharge but not survival of arrest. Respiratory arrest was an independent predictor of survival, but there was no association between respiratory arrest and bradycardic PEA arrest.

## 1 Introduction

There are estimated to be approximately 200,000 in-hospital cardiac arrests (IHCA) each year in the United States [1], and this number is likely growing [2, 3]. Mortality for patients with IHCA remains high, with overall survival rates ranging from 17-22% but even lower for those with pulseless electrical activity (PEA) arrest or asystole [4]. Fortunately, survival to discharge in patients with IHCA has increased over the past two decades [2].

Few studies have assessed the outcomes of patients with bradycardia prior to arrest. Bradycardia may be common in patients with in-hospital cardiac arrest and may predict worse patient outcomes in patients outside of the intensive care unit [5]. Bradycardia has been demonstrated to precede respiratory arrest in canine and porcine asphyxiation models [6, 7]. In the pediatric population, bradycardia is often regarded as a marker of impending respiratory arrest and frequently precedes pulseless electrical activity (PEA) arrest. Children who receive chest compressions with bradycardia and poor perfusion have improved survival to hospital discharge compared to those who receive chest compressions for PEA arrest.

Certain clinical or patient variables around or at the time of cardiac arrest may play a role in identifying patients with improved outcomes. For instance, clinical extremes in respiratory rate, pulse rate, blood pressure, and oxygenation have been associated with poor patient outcomes following arrest [8, 9]. Many studies have attempted to identify clinical variables that predict whether a patient will develop cardiorespiratory arrest and survival to discharge, but few studies have investigated the role of bradycardia in adults with IHCA and whether bradycardia is a marker of patient outcomes.

There continues to be a dearth of data on the relationship between bradycardia and respiratory arrest, and whether the presence of either is associated with patient outcomes in those with IHCA. The purpose of this study is to determine the prevalence of bradycardia at the time of cardiac arrest in patients with pulseless electrical activity, and to determine the relationship between bradycardia and respiratory arrest in adult, hospitalized patients.

## 2 Methods

### 2.1 Design

The study is a retrospective cohort analysis of a single 450-bed tertiary care center in an urban setting. Eligible patients were identified from the in-hospital cardiac arrest database, starting from July 2013 at the time of the database's creation through August 2017. The hospital includes both teaching and non-teaching services. The study was approved by the University of Washington Institutional Review Board via an expedited review.

### 2.2 Identification of study participants

Patients were eligible for inclusion in the study if they were ≥18 years old and had a cardiac arrest activation while physically present in the hospital. Participants were identified from the in-hospital database of information on all cardiac arrest activations. Study patients included those hospitalized both in general medical/surgical acute care and intensive care beds. Patients with code blue activation in the emergency department, radiology suite, procedural areas, and operating rooms were not included in the study. In addition, those with vasovagal reactions and incomplete, uninterpretable, or contradictory initial rhythm data were excluded.

### 2.3 Variables and measurements

Once eligible patients were identified from the cardiac arrest database, demographic data and event data were abstracted from the electronic hospital medical record. Please refer to supplemental appendix for complete list of variables obtained from cardiac arrest documentation.

We defined a pulseless electrical activity arrest as a cardiac arrest with the initial rhythm as non-shockable with organized electrical activity requiring ≥1 round of chest compressions. We defined a bradycardic PEA arrest as a PEA arrest with bradycardia at the time of loss of pulse. Patients were considered to have bradycardia at the time of pulselessness if they had a QRS frequency of less than 60 beats/min on telemetry, were noted by first responders to be bradycardic with a pulse rate of less than 60 beats/min prior to cardiac arrest, or if the term “bradying down,” was identified in any of the cardiac arrest event notes.

Two independent investigators (D.N. and J.M.P.) reviewed the arrests to determine whether bradycardia was present at the onset of pulselessness. The determination was made using a combination of first responder notes, nursing notes, code blue summary notes, death notes, vital signs, and telemetric data when available. In addition, each investigator independently reviewed each code event, death note, and discharge summary in conjunction with objective data and attributed a potential etiology, such as respiratory failure, myocardial infarction, or shock.

### 2.4 Primary endpoints

The primary endpoints were survival of cardiac arrest event and survival to hospital discharge.

### 2.5 Statistics

We performed descriptive analysis using student's T-test for continuous variables and chi-squared analysis for discrete, binary, categorical variables. The alpha level for statistical significance was α = 0.05. All statistical analyses were conducted through commercially available software (Wizard, version 1.9.32).

We used univariate and multivariate regression models to determine the impact of bradycardia at the time of PEA arrest on the primary endpoint. Prospectively designated, clinically important potential confounders [age, gender, ethnicity, medical comorbidities, home medications, reason for hospitalization, hospital service in which care was received (medical/surgical, intensive care, cardiac), medications received up to 48 h prior to arrest, whether the code was witnessed, whether the patient was on a monitored unit, vital signs up to 4 h prior to arrest, rhythm at onset of pulselessness, time of intubation, and therapies received during the code] were abstracted from the medical record and included as candidate predictors in the models. These patient factors were included in the multivariate model if there was a univariate difference with a P value of ≤0.10.

## 3 Results

During the study period from July 2013 through August 2017, there were 257 in-hospital PEA arrests recorded in 190 individuals. For the 67 patients who had repeat events, we only included the first cardiac arrest in the analysis. Each code was analyzed as an individual event with regards to the both primary outcomes. One patient was excluded for being an out of hospital cardiac arrest that continued to be resuscitated in the ED. Two code events had incomplete, incorrect, or uninterpretable data and were removed. Lastly, there were 11 clearly-defined vasovagal events for which code blue was activated. After removing these patients, there were 176 patients with PEA arrest with interpretable, complete data on their code events.

**Figure 1:**
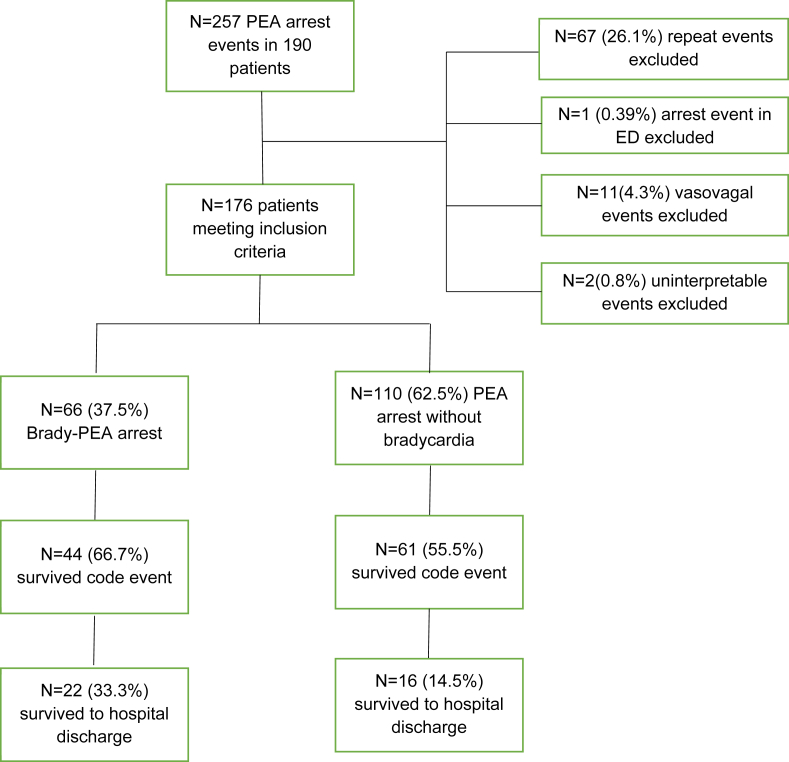
Flowchart of patients meeting inclusion criteria.

Of the 176 patients, 71 (40.3%) did not achieve return of spontaneous circulation. 105 (59.7%) patients survived the initial code event, but 67 (38.1%) of those patients died in the hospital while 38 (21.6%) survived to hospital discharge (Figure 1). In total, 66 (37.5%) of included patients had a bradycardic PEA arrest and 110 (62.5%) had a non-bradycardic- PEA arrest. Of the bradycardic PEA group, 44 (66.7%) survived to hospital discharge while 22 (33.3%) survived to hospital discharge in the non-bradycardic PEA group. Three (4.5%) patients with bradycardic PEA arrests had a temporary pacing wire placed and 2 (3.0%) received a permanent pacemaker during their hospitalization.

Between the bradycardic PEA group and the non-bradycardic PEA group, there was no significant difference with respect to age, ethnicity, gender, obesity status, or co-morbid conditions including coronary artery disease, lung disease, or renal disease (Table 1). Patients with bradycardic PEA arrest also had no difference in the rate of beta blockers, calcium channel blockers, or antiarrhythmic drug use in the 48 h prior to cardiac arrest. Patients with non-bradycardic PEA arrests were more likely to have a CIED than bradycardic PEA patients (14.5% vs 3.0%, P = 0.02).

**Table 1:** Brady-PEA vs non-Brady PEA arrests. Items in bold have P-value <0.05.

	Non-brady PEA (n = 110)	Brady-PEA (n = 66)	P-value
Age (mean ± SD)	61.7 ± 14.6	58.8 ± 15.1	0.21
Female Sex	35 (31.8%)	18 (27.3%)	0.61
Coronary Artery Disease	31 (28.8%)	21 (31.8%)	0.61
**Cardiac implantable electronic device**	**16 (14.5%)**	**2 (3.0%)**	**0.02**
Obesity	31 (28.4%)	19 (28.8%)	1.0
Monitored Unit (n = 146)	96 (87.3%)	34 (94.4%)	0.36
Witnessed	96 (87.3%)	60 (90.1%)	0.63
Heart Failure	29 (26.4%)	18 (27.3%)	1.0
Beta Blocker	25 (22.7%)	21 (31.8%)	0.22
Calcium Channel Blocker	7 (6.4%)	3 (4.5%)	0.75
Antiarrhythmic Drug	16 (14.5%)	13 (19.7%)	0.41
Renal Disease	21 (19.1%)	11 (16.7%)	0.84
Lung Disease	32 (29.1%)	24 (36.4%)	0.32
**Cause**			**0.006**
Acidemia	5 (4.5%)	1 (1.5%)	
Cardiac tamponade	4 (3.6%)	3 (4.5%)	
Electrolyte ab normality	3 (2.7%)	3 (4.5%)	
Respiratory	30 (27.3%)	24 (36.4)	
Myocardial infarction	4 (3.6%)	7 (10.6%)	
Pulmonary embolus	5 (4.5%)	4 (6.1%)	
Shock	36 (32.7%)	8 (12.1%)	
Other	10 (9.1%)	11 (16.7%)	
Unknown	13 (11.8%)	2 (3.0%)	
Respiratory arrest	30 (27.3%)	24 (36.4%)	0.24
Intubated prior to code	38 (34.5%)	15 (22.7%)	0.12

Bradycardic PEA patients were no more likely to have their arrest precipitated by respiratory arrest than non-bradycardic PEA patients (36.4% vs 27.3%, P = 0.24), and there was no differences between groups with regards to intubation before code or within 15 min of code onset (22.7% vs 34.5%, P = 0.12). Intubation before code or within 15 min did not have an impact on survival of code or to hospital discharge in either the bradycardic PEA group or non-bradycardic PEA group (Table 2). However, there were significant differences in survival of code and to discharge in the bradycardic PEA group and non-bradycardic PEA group with respect to cause of arrest. Patients with bradycardic PEA were less likely to have shock and more likely to have respiratory arrest as the cause for arrest, with patients in the shock cohort less likely to survive and patients with respiratory arrest more likely to survive (Tables 1 and 2).

**Table 2:** Primary outcomes and associated clinical variables of interest. Items in bold have P value <0.05

	Survival of Code (n = 105)	Died at Code (n = 71)	P-Value	Survival to Discharge (n = 38)	Died before Discharge (n = 139)	P-value
Age (mean ± SD)	60.2 ± 14.5	61.2 ± 15.4	0.65	60.3 ± 15.8	60.7 ± 14.6	0.87
Female Sex	31 (29.5%)	22 (31.0%)	0.87	8 (21.1%)	45 (32.6%)	0.23
Coronary Artery Disease (CAD)	30 (28.6%)	22 (31.0%)	0.74	16 (42.1%)	36 (26.1%)	0.07
Cardiac implantable electronic device	6 (8.5%)	12 (11.4%)	0.62	5 (13.2%)	13 (9.4%)	0.55
Obesity	**36 (34.3%)**	**14 (20.0%)**	**0.04**	13 (34.2%)	37 (27.7%)	0.42
Monitored Unit (n = 146)	74 (88.1%)	56 (90.3%)	0.79	26 (92.9%)	104 (88.1%)	0.74
Witnessed	95 (90.5%)	61 (85.9%)	0.47	**38 (100%)**	**118 (85.5%)**	**0.008**
Heart Failure	26 (24.8%)	21 (30.0%)	0.49	11 (28.9%)	36 (26.1%)	0.84
Beta Blocker	17 (23.9%)	29 (27.6%)	0.61	37 (26.8%)	9 (23.7%)	0.84
Calcium Channel Blocker	5 (7.0%)	5 (4.8%)	0.53	9 (6.5%)	1 (2.6%)	0.69
Antiarrhythmic Drug	11 (15.5%)	18 (17.1%)	0.84	24 (17.4%)	5 (13.2%)	0.63
Renal Disease	18 (17.1%)	14 (19.7%)	0.69	6 (15.8%)	26 (18.8%)	0.81
Lung Disease	38 (36.2%)	18 (25.4%)	0.14	13 (34.2%)	43 (31.2%)	0.70
Respiratory arrest	**41 (39.0%)**	**13 (18.3%)**	**0.01**	**19 (50.0%)**	**35 (25.2%)**	**0.005**
Causes			**0.001**			**<0.0001**
Acidemia	**5 (4.7%)**	**1 (1.4%)**		**0 (0%)**	**6 (4.3%)**	
Cardiac tamponade	**2 (1.9%)**	**5 (7.0%)**		**0 (0%)**	**7 (5.0%)**	
Electrolyte abnormality	**4 (3.6%)**	**2 (2.8%)**		**0 (0%)**	**6 (4.3%)**	
Respiratory	**41 (39.0%)**	**13 (18.3%)**		**19 (50.0%)**	**35 (25.2%)**	
Myocardial infarction	**6 (5.5%)**	**5 (7.0%)**		**3 (7.9%)**	**8 (5.7%)**	
Pulmonary embolus	**3 (2.7%)**	**6 (8.5%)**		**1 (2.6%)**	**8 (5.7%)**	
Shock	**19 (17.3)**	**25 (35.2%)**		**2 (5.2%)**	**42 (30.2%)**	
Other	**18 (16.4%)**	**3 (4.2%)**		**11 (28.9%)**	**10 (7.2%)**	
Unknown	**10 (9.5%)**	**5 (7.0%)**		**2 (5.2%)**	**13 (9.4%)**	
Brady-PEA	44 (41.9%)	22 (31.0%)	0.16	**22 (57.9%)**	**44 (31.9%)**	**0.0045**
Vasopressin	17 (16.2%)	22 (31.0%)	0.25	5 (13.2%)	34 (24.4%)	0.18
Epinephrine	**93 (88.6%)**	**70 (98.5%)**	**0.003**	**29 (76.3%)**	**134 (96.4%)**	**<0.001**
Atropine	17 (16.2%)	16 (22.5%)	0.33	4 (10.5%)	29 (20.9%)	0.23
Sodium bicarbonate	**65 (61.9%)**	**60 (84.5%)**	**0.001**	**16 (42.1%)**	**109 (78.4%)**	**<0.0001**
Intubated prior to code or within 15 min of onset	76 (72.3%)	55 (77.5%)	0.49	26 (68.4%)	105 (76.0%)	0.40

Univariate analysis and multivariate analyses are included in Tables 3 and 4 for survival of code and survival to hospital discharge, respectively. On multivariate analysis, bradycardic PEA was associated with improved survival to hospital discharge (OR = 3.31, 95% CI: 1.41–7.79, p = 0.006), but not survival of arrest (OR 1.45, 95% CI: 0.68–3.09, P = 0.34). Respiratory arrest was an independent predictor of survival of code (OR 2.62, 95% CI: 1.36–5.47, P = 0.01) and to hospital discharge (OR 3.47, 95% CI: 1.35–8.91, P = 0.01).

**Table 3:** Univariate and multivariate predictors of survival of code. Items in bold have P value <0.05

Variable	Univariate			Multivariate		
	Odds Ratio	95% Confidence Interval	P-value	Odds Ratio	95% Confidence Interval	P-value
Brady-PEA	**2.00**	**1.20–3.34**	**0.008**	1.45	0.68–3.09	0.34
Respiratory arrest	**3.15**	**1.69–5.89**	**<0.001**	**2.62**	**1.36–5.47**	**0.01**
Obesity	**2.57**	**1.39–4.77**	**0.003**	**2.30**	**1.09–4.86**	**0.03**
Witnessed	**1.56**	**1.13–2.15**	**0.007**	**1.97**	**1.06–3.66**	**0.03**
Lung Disease	**2.11**	**1.21–3.70**	**0.009**	1.79	0.86–3.71	0.12
Cardiac implantable electronic device	2.00	0.75–5.33	0.17	2.12	0.56–8.10	0.27
Heart Failure	1.24	0.70–2.20	0.47	0.54	0.24–1.18	0.12
Beta Blocker	1.76	0.94–3.10	0.08	1.50	0.67–3.36	0.33
Antiarrhythmic Drug	1.636	0.77–3.47	0.20	2.84	0.92–8.79	0.07
Coronary Artery Disease	1.36	0.79–2.36	0.27	1.34	0.59–3.04	0.49
Monitored Unit	1.32	0.93–1.87	0.12	0.67	0.22–2.04	0.49
Vasopressin	0.77	0.41–1.46	0.43	0.65	0.29–1.46	0.30
Epinephrine	**1.33**	**0.94–1.81**	**0.07**	0.74	0.18–3.12	0.68
Atropine	1.06	0.54–2.10	0.86	0.64	0.28–1.46	0.28
Sodium bicarbonate	1.08	0.76–1.54	0.66	**0.38**	**0.20–0.71**	**0.003**
Intubated prior to code or within 15 min of onset	1.38	0.98–1.96	0.07	1.11	0.49–2.51	0.79

**Table 4:** Univariate and multivariate predictors of survival to hospital discharge. Items in bold have P value <0.05

Variable	Univariate Analysis			Multivariate Analysis		
	Odds Ratio	95% confidence interval	P-value	Odds Ratio	95% confidence interval	P-value
Brady-PEA	**2.94**	**1.41–6.20**	**0.004**	**3.31**	**1.41–7.78**	**0.006**
Coronary Artery Disease	2.06	0.98–4.35	0.058	**3.25**	**1.28–8.30**	**0.013**
Respiratory arrest	**3.63**	**1.63–8.08**	**0.002**	**3.47**	**1.35–8.91**	**0.01**
Cardiac implantable electronic device	1.46	0.49–4.38	0.50	1.85	0.29–11.94	0.52
Heart Failure	1.15	0.52–2.56	0.72	0.55	0.16–1.91	0.35
Beta Blocker	**0.24**	**0.12–0.50**	**<0.001**	0.52	0.16–1.69	0.27
Antiarrhythmic Drug	**0.21**	**0.08–0.55**	**0.001**	1.09	0.13–9.33	0.94
Witnessed	6.12	0.79–47.24	0.08	4.19	0.82–21.36	0.08
Monitored Unit	1.75	0.38–8.19	0.48	1.31	0.17–10.16	0.80
Obesity	1.41	0.65–3.03	0.38	0.99	0.27–3.72	0.99
Lung Disease	1.15	0.54–2.46	0.72	0.98	0.33–2.87	0.96
Vasopressin	**0.15**	**0.058–0.38**	**<0.001**	0.70	0.21–2.39	0.57
Epinephrine	**0.22**	**0.15–0.32**	**<0.001**	**0.14**	**0.05–0.37**	**<0.001**
Atropine	**0.14**	**0.048–0.39**	**<0.001**	**0.21**	**0.055–0.81**	**0.023**
Sodium bicarbonate	**0.15**	**0.09–0.25**	**<0.001**	**0.33**	**0.13–0.86**	**0.023**
Intubated prior to code or within 15 min of onset	**0.25**	**0.16–0.38**	**<0.001**	2.62	0.79–8.74	0.12

Additional factors associated with survival of code included witnessed arrest, obesity, and lack of lack of treatment with sodium bicarbonate, while factors associated with survival to hospital discharge included the presence of coronary artery disease and lack of treatment with epinephrine, sodium bicarbonate, or atropine.

## 4 Discussion

In this retrospective study, we demonstrated that patients with bradycardic PEA arrests had an increased survival to hospital discharge compared to those with non-bradycardic PEA arrest. We found no relationship between bradycardic PEA arrests and respiratory arrest, unlike in pediatric patients where bradycardia usually precedes respiratory arrest [10]. Respiratory arrest as the cause of PEA increased the likelihood of survival of code and survival to hospital discharge independent of whether or not there was bradycardia prior to PEA. To our knowledge, our study is the first to study the relationship between bradycardia prior to cardiac arrest and respiratory arrest in human patients, as this phenomenon has previously only been described in animal studies [6, 7].

The rates of survival to hospital discharge in patients with PEA arrest in our study were nearly double that of historical controls, with a survival rate of 21.6% [2]. The proportion of patients who survive to hospital discharge after PEA arrest nation-wide is estimated to be 10–11%, but this proportion is increasing yearly due to the adoption of hospital wide recording and reporting cardiac arrest quality improvement metrics, better post-resuscitation care, and earlier recognition of cardiac arrest [2]. In our study, bradycardic PEA arrests had an increased survival to hospital discharge compared to non-bradycardic PEA patients. There are no available historical controls to compare this to, as no studies to our knowledge make the distinction of non-bradycardic PEA and bradycardic PEA arrests. Our results demonstrate that bradycardic PEA patients have a different risk profile, etiology of arrest, and survival than non-bradycardic PEA patients, and perhaps more time should be spent resuscitating these patients due to their higher survival rate. Overall, a small number of patients with bradycardic PEA arrest went on to receive a temporary pacing wire or permanent pacemaker.

Bradycardia prior to PEA was an independent predictor of survival to hospital discharge, but may not be the only reason that these patients were more likely to survive. These patients were less likely to receive epinephrine, atropine, and sodium bicarbonate suggesting they may have been a healthier cohort. It is possible that some of these patients had extensive vasovagal episodes requiring resuscitation, and we attempted to exclude these patients as best as possible based on the data available. Additional factors including frailty, type of bed, day of week, time of cardiac arrest, and duration of resuscitation [11] are all factors that may influence cardiac arrest outcomes in this cohort but were not available. This is an area we have targeted for future study.

The heart rate cutoff of 60 beats per minute for bradycardic PEA patients was chosen to mirror a previous study that assessed studied outcomes of patients with bradycardia prior to VT/VF arrests [5]. The incidence of bradycardia prior to PEA arrest in our cohort was 37.5%, while the incidence of bradycardia prior to VT/VF arrest was 54.1% in the prior study [5], and we suspect that it is quite common prior to cardiac arrest. The true incidence of bradycardia prior to IHCA in both VT/VF and PEA/asystole is unknown, however, as no studies have detailed this in a large, multicenter cohort study. Perhaps pre-arrest rhythm should be included as a variable in IHCA databases to further delineate the impact on pre-arrest rhythm on survival. Lastly, it is unclear if additional electrocardiographic factors, such as PR interval, QRS duration, QT duration, and the presence of high-grade block contribute to outcomes in this cohort and may be an area of further investigation.

The physiologic heart rate response to cardiac arrest may play a role in patient outcomes. Current data on survival outcomes in patients with bradycardia prior to cardiac arrest are limited to one other small retrospective study. Bhalala et al demonstrated that bradycardia within 10 min preceding cardiac arrest resulted in increased risk of death prior to hospital discharge in patients with both PEA arrest and ventricular tachyarrhythmias in ICU [5]. Our study contributes to this knowledge gap in the form of a study with a larger population, but our primary outcome data conflict with that of Bhalala et al. [5] Possible explanations include differences in how our institution manages patients with bradycardia or different patient populations, as theirs included only patients in intensive care. Our population included a broad range of medical specialties, ranging from patients in the bone marrow transplant unit to those with in the coronary care unit. Besides pre-arrest bradycardia, there is increasing evidence that post-arrest bradycardia may influence outcomes. Recent data from Thomsen et al suggested that bradycardia during targeted temperature management for out-of-hospital cardiac arrest was an independent predictor of survival to discharge and survival with good neurologic outcome [12].

Animal models of cardiac arrest precipitated by asphyxiation show that profound bradycardia followed by hemodynamic collapse is the common pathway to PEA/asystolic arrests [6, 7], but our study did not demonstrate an association with hypoxemia and bradycardia in adults. Furthermore, intubation within the first 15 min of the code did not impact survival of code or to hospital discharge. Recent studies assessing the impact of early intubation versus bag-mask ventilation in cardiac arrest patients demonstrated similar results. A recent large-scale observational study by Andersen et al using American Heart Association Get with the Guidelines-Resuscitation registry demonstrated a relative 9% reduction in survival in patients with PEA arrest [13], while a randomized controlled trial by Jabre et al did not demonstrate non-inferiority or inferiority between bag-mask ventilation and tracheal intubation [14]. While our multivariate model was likely underpowered to detect differences and may include confounding factors such as indication for intubation, our findings suggest that there is no benefit to early tracheal intubation, and perhaps high-quality chest compressions should be considered a priority in this cohort.

Our study has a number of limitations. Our results must be considered in the context of the study's observational nature, and confounding factors such as quality of chest compressions, patient frailty, time of day, and day of week could not be completely controlled for. Thus, our results should be considered as hypothesis generating. While the cardiac arrest response staff undergo rigorous training for proper documentation and recording of arrest events, data integrity and viability must be considered as a potential confounder in this retrospective study. We did not have complete objective data on telemetry, respiratory rate and oxygen saturation at the time of cardiac arrest, and the labels of bradycardic-PEA and hypoxemia were assigned by cardiac arrest staff at the time of the event. This was interpreted in the context of the cardiac arrest by the attending physician, and we acknowledge this as a possible source of recording bias. We attempted to mitigate this by removing code events from the final data set without interpretable code data or a clear cause for arrest. Next, the sample size was small, which may have limited our ability to detect significant differences between the two exposure groups in our cohort. Our patients were recruited from a single tertiary academic medical center, limiting the ability to generalize conclusions to the general public. Lastly, our study did not include survival with good neurologic outcome as a primary endpoint, as these data were not available.

## 5 Conclusion

In a retrospective study of hospitalized patients in the intensive care unit and non-intensive care, bradycardia at the time of cardiac arrest was associated with improved survival to hospital discharge but not survival of arrest. Respiratory arrest was an independent predictor of survival, but there was no association between respiratory arrest and bradycardic-PEA arrest.

## Declarations

### Author contribution statement

D. Nguyen: Conceived and designed the experiments; Analyzed and interpreted the data; Wrote the paper.

P. Kritek and S. Greco: Analyzed and interpreted the data; Contributed reagents, materials, analysis tools or data; Wrote the paper.

J. Prutkin: Conceived and designed the experiments; Analyzed and interpreted the data; Contributed reagents, materials, analysis tools or data; Wrote the paper.

### Funding statement

This research did not receive any specific grant from funding agencies in the public, commercial, or not-for-profit sectors.

### Competing interest statement

The authors declare no conflict of interest.

### Additional information

No additional information is available for this paper.

## Appendix A Supplementary data

Resus Paper Supplemental Appendix Resus Paper Supplemental Appendix

## References

[1] Merchant R.M., Yang L., Becker L.B. (2011). Incidence of treated cardiac arrest in hospitalized patients in the United States. Crit. Care Med..

[2] Girotra S., Nallamothu B.K., Spertus J.A., Li Y., Krumholz H.M., Chan P.S. (2012). Trends in survival after in-hospital cardiac arrest. N. Engl. J. Med..

[3] Andersen L.W., Holmberg M.J., Berg K.M. (2019). In-Hospital Cardiac Arrest: A Review. JAMA.

[4] Peberdy M.A., Kaye W., Ornato J.P. (2003). Cardiopulmonary resuscitation of adults in the hospital: a report of 14 720 cardiac arrests from the National Registry of Cardiopulmonary Resuscitation. Resuscitation.

[5] Bhalala U.S., Bonafide C.P., Coletti C.M. (2012). Antecedent bradycardia and in-hospital cardiopulmonary arrest mortality in telemetry-monitored patients outside the ICU. Resuscitation.

[6] Debehnke D.J., Hilander S.J., Dobler D.W., Wickman L.L., Swart G.L. (1995). The hemodynamic and arterial blood-gas response to asphyxiation - a canine model of pulseless electrical-activity. Resuscitation.

[7] Lee A., Luong D., Lee T., Reilly M.O., Cheung Y., Schmo G.M. (2019). Non-perfusing cardiac rhythms in asphyxiated newborn piglets. PloS One.

[8] Andersen L.W., Young W., Chase M. (2016). The prevalence and significance of abnormal vital signs prior to in-hospital cardiac arrest.

[9] Rozen T.H., Mullane S., Kaufman M. (2014). Antecedents to cardiac arrests in a teaching hospital intensive care unit. Resuscitation.

[10] Donoghue A., Berg R.A., Hazinski M.F. (2009). Cardiopulmonary resuscitation for bradycardia with poor perfusion versus pulseless cardiac arrest. Pediatrics.

[11] Graham R., McCoy M.A., Schultz A.M. (2015 Sep 29). Strategies to improve cardiac arrest survival: a time to act. In-Hospital Cardiac Arrest and Post-Arrest Care.

[12] Thomsen J.H., Nielsen N., Hassager C. (2016). Bradycardia during targeted temperature management: an early marker of lower mortality and favorable neurologic outcome in comatose out-of-hospital cardiac arrest patients. Crit. Care Med..

[13] Andersen L.W., Granfedlt A., Callaway C.W. (2017). Association between tracheal intubation during adult in-hospital cardiac arrest and survival. J. Am. Med. Assoc..

[14] Jabre P., Penaloza A., Pinero D. (2018). Effect of bag-mask ventilation vs endotracheal intubation during cardiopulmonary resuscitation on neurological outcome after out-of-hospital cardiorespiratory arrest: a randomized clinical trial. J. Am. Med. Assoc..

