# Supplementary material for: Bradycardia at the onset of pulseless electrical activity arrests in hospitalized patients is associated with improved survival to discharge
Source: Heliyon. 2020 Feb 28;6(2):e03491. doi: 10.1016/j.heliyon.2020.e03491 (PMC7049649; doi:10.1016/j.heliyon.2020.e03491)
Supplement: Resus Paper Supplemental Appendix [file mmc1.docx]

*Bradycardia at the Onset of Pulseless Electrical Activity Arrests in Hospitalized Patients is Associated with Improved Survival to Discharge*

Supplemental Appendix: Medical Emergency Response Committee Code Blue Variables

| Time of Code | *Type of Arrest* | *Intubation Status* | *Rhythm At Onset* | *Breathing at Onset* | *Reason Ended* | *Patient Outcome* |
| --- | --- | --- | --- | --- | --- | --- |
| Date of code | Cardiac | Not Intubated | VT/VF | Spontaneous | Return of Spontaneous Circulation | Deceased |
| Timekeeping source | Respiratory | Intubated before code | PEA | Assisted | No ROSC | Remained in Unit |
| Code End Time | Anaphylaxis | Intubated during code | Asystole | Agonal | Advance Directive | Transferred to: |
| Witnessed arrest? | Not an Arrest | Time of Code Intubation | Bradycardia | Apneic | Family Request |  |
|  |  | No. of attempts | NSR | Intubated/ETT | Other |  |
|  |  | Type of airway: ETT vs surgical | Other |  |  |  |
|  |  | ETT confirmation: EtCO2 vs auscultation |  |  |  |  |

|  | Time↦ |  |  |  |  |  |
| --- | --- | --- | --- | --- | --- | --- |
| *Code Variables* |  |  |  |  |  |  |
| Pulse (Y/N) |  |  |  |  |  |  |
| Rhythm Name and Rate |  |  |  |  |  |  |
| Compressions (Y/N) |  |  |  |  |  |  |
| EtCO2 |  |  |  |  |  |  |
| Shock (C= cardiovert, D = defib |  |  |  |  |  |  |
| Pacing? (Temp or permanent) |  |  |  |  |  |  |
| Airway management (BMV, ETT) |  |  |  |  |  |  |
| O2 Sat |  |  |  |  |  |  |

| *Meds + dose* | Time↦ |  |  |  |  |  |
| --- | --- | --- | --- | --- | --- | --- |
| Epinephrine |  |  |  |  |  |  |
| Vasopressin |  |  |  |  |  |  |
| Amiodarone |  |  |  |  |  |  |
| Lidocaine |  |  |  |  |  |  |
| Atropine |  |  |  |  |  |  |
| IV Fluids |  |  |  |  |  |  |
| Blood Products |  |  |  |  |  |  |
| Dopamine Drip |  |  |  |  |  |  |
| Epinephrine Drip |  |  |  |  |  |  |
| Norepinephrine Drip |  |  |  |  |  |  |
| Other |  |  |  |  |  |  |

Referred to in paper as Supplemental Appendix.
